# Successful treatment of thrombotic thrombocytopenic purpura with plasmapheresis and anti-CD20 antibodies in a patient with immune thrombocytopenia and systemic lupus erythematosus: Case report

**DOI:** 10.1097/MD.0000000000028908

**Published:** 2022-02-18

**Authors:** Ju-Yang Jung, Ji-Won Kim, Chang-Hee Suh, Hyoun-Ah Kim

**Affiliations:** Department of Rheumatology, Ajou University of Medical School, Suwon, Korea.

**Keywords:** case report, immune thrombocytopenia, rituximab, systemic lupus erythematosus, thrombotic Thrombocytopenic purpura

## Abstract

**Rationale:**

Systemic lupus erythematosus (SLE) is an autoimmune disease of unknown etiology with diverse clinical and laboratory manifestations, including thrombocytopenia. About 25% of patients with SLE may be affected by thrombocytopenia, many of whom are asymptomatic. Some patients, however, experience platelet counts that drop quite low and predispose them to bleeding. Thrombotic thrombocytopenic purpura (TTP) is defined with a classic pentad of clinical features, such as thrombocytopenia, microangiopathic hemolytic anemia, neurological symptoms and signs, renal symptoms and signs, and fever. The association of TTP and SLE has been sporadically reported in the literature.

**Patient concerns and diagnosis:**

We describe a 16-year-old girl with SLE and immune thrombocytopenia, in whom TTP was diagnosed.

**Interventions and outcomes:**

She was treated with pulse methylprednisolone, whose platelet counts normalized after therapy with plasmapheresis and an anti-CD20 monoclonal antibody (rituximab).

**Conclusion:**

A pediatric patient with SLE and immune thrombocytopenia in whom TTP developed was treated with plasmapheresis and rituximab therapy successfully, though the patient experienced a disease relapsed after 18 months, which was controlled by the same management.

## 1 Introduction

Thrombotic thrombocytopenic (TTP) is a rare and life-threatening disorder characterized by microangiopathic hemolytic anemia with thrombocytopenia, fever, renal changes, and neurological symptoms.^[^1^,^^2^^]^ While the pathogenesis of TTP is not understood yet, a deficiency of von Willebrand factor (VWF) cleaving protein, also known as a disintegrin and metalloproteinase with a thrombospondin type 1 motif, member 13–VWF cleaving protein (ADAMTS13) causes TTP.^[^3^,^^4^^]^ Congenital TTP is caused by an inherited deficiency of ADAMTS13 and acquired TTP is caused by autoantibodies against ADAMTS13. Uncleaved, large, VWF multimers formed due to a defect in ADAMTS13 leads to spontaneous platelet aggregates in the microvasculature of the brain, heart, and kidneys. Acquired TTP, also called as immune-mediated TTP, can occur without any precipitating cause, or secondary to other autoimmune diseases including systemic lupus erythematosus (SLE).^[5]^

The identification of microangiopathic hemolytic anemia and thrombocytopenia without alternative etiology are essential in the diagnosis of TTP.^[^6^,^^7^^]^ Clinical manifestations derive from the involved organs, including the central nervous and gastrointestinal systems. Neurologic symptoms include difficulty speaking, numbness, weakness, seizure, and mental state changes, whereas gastrointestinal symptoms include abdominal pain, diarrhea, nausea, and vomiting. With such clinical features, it is important to confirm a defect in ADAMTS13 activity in order to diagnose TTP.^[8]^ It can be difficult to await confirmation of ADAMTS13 results before starting the management of TTP in emergent situations. Prompt plasmapheresis is the gold standard treatment in patients with TTP, often in combination with immunosuppressive agents.

SLE is an autoimmune disease in which organs, tissues, and cells undergo damage mediated by tissue-binding autoantibodies and immune complexes. Hematologic manifestations are common in SLE; leukopenia occurs in 50% to 60%, and thrombocytopenia occurs in 25% of patients with SLE.^[^9^,^^10^^]^ TTP rarely develops in patients with SLE, though, and outcomes are known to be similar to those of primary immune TTP.^[^11^,^^12^^]^

We describe a pediatric patient with SLE who manifested TTP, was treated successfully with plasmapheresis and rituximab, and experienced a TTP relapse after 18 months that remitted after the same management.

## 2 Case report

A 16-year-old girl was admitted for whole body petechiae that began 2 days prior to admission, and headache, dizziness, and left arm and leg weakness that began that day. Relevant past medical history included presenting to the pediatric outpatient clinic with whole body petechiae and thrombocytopenia one year prior in September 2018. She was referred to rheumatology for leukopenia (2500/μL), thrombocytopenia (78,000/μL), positive anti-nuclear antibody (>1:2560, speckled type), a positive anti-double stranded (ds)DNA antibody (25.8 IU/mL), hypocomplementemia (C3: 53 mg/dL, C4: 11 mg/dL), alopecia, and malar rash. She was diagnosed with SLE. After treatment with hydroxychloroquine and a low dose steroid (6 mg methylprednisolone), her symptoms resolved, but whole body petechiae and menorrhagia recurred in April 2019, and severe thrombocytopenia (8000/μ) developed without anemia (Hb 12.4 g/dL) or leukopenia (3500/μL). A bone marrow biopsy was done and showed normocellular marrow. She recovered with high dose steroid and intravenous immunoglobulin treatments.

On the admission day (June 2019), the patient was acutely ill-looking but had no fever. She was normotensive (BP 102/52 mm Hg) and her pulse rate was 99/minute. Bilateral leg petechiae was noted. There was no cervical lymphadenopathy or hepatosplenomegaly. She did not have joint pains, oral ulcers, malar rash, or photosensitivity. Laboratory studies at this time showed a hemoglobin level of 7.9 g/dL, a white blood cell count of 10,800/uL, a platelet count of 8000/uL, erythrocyte sedimentation rate of 29 mm/h, C-reactive protein 0.13 mg/dL, and total bilirubin of 4.8 mg/dL. Urinalysis revealed one red blood cell per high-power field and negative proteinuria. C3 and C4 levels were 82 mg/dL and 6 mg/dL, respectively. Anti-double stranded DNA antibody was in the normal range. Brain magnetic resonance imaging did not show any parenchymal lesion or perfusion abnormalities. Peripheral blood smear indicated anisocytosis, poikilocytosis, and the presence of schistocytes, and ADAMTS13 activity was low at 16%.

In view of TTP with low SLE activity, hydroxychloroquine, methylprednisolone 1 g pulse, IVIG 400 mg/kg for 5 days and plasmapheresis for 5 days were prescribed. The patient's platelet count increased to 231,000/uL. Four cycles of rituximab were administered weekly with high dose glucocorticoids. After 4 cycles of rituximab therapy, her platelet count had increased to 305,000/uL. Therefore, the glucocorticoids dose was reduced gradually with the addition of tacrolimus 2 mg. The platelet count increased to 337,000/uL 2 months later. After 1 year and 2 months, the patient developed headache with severe thrombocytopenia at 10,000/uL. ADAMTS activity was 0.5%. Four cycles of rituximab were administered weekly with plasmapheresis for 5 days. Thrombocytopenia recovered to 298,000/uL. Presently, she takes low dose prednisolone (daily 5 mg) and hydroxychloroquine (daily 300 mg) and her platelet count is maintained at more than 200,000/uL. Table 1 shows changes of platelet counts associated with ITP or TTP.

**Table 1:** Changes of platelet counts according to various treatments.

	1st immune thrombocytopenia (14/SEP/2018)	2nd immune thrombocytopenia (5/APR/2019)	1st TTP (02/JUN/2019)	2nd TTP (25/NOV/2020)
Clinical manifestations				
Fever	+	+	+	-
Malar rash	+	-	-	-
Oral ulcer	-	-	-	-
Arthritis	-	-	-	-
Serositis	-	-	-	-
Laboratory data				
WBC, /μL	2500	3500	10,800	5,700
Hb, gd/L/Hct %	9.6/28.3	11.6/33.1	7.9/22.8	9.4/26.8
Platelets, ×1,000/μL	78	8	8	7
ESR, mm/hr	28	11	9	8
CRP, mg/dL	3.39	0.08	0.13	0.06
Total bilirubin, mg/dL	0.7	1.4	4.8	3.2
AST, IU/L	73	25	91	29
ALT, IU/L	63	15	17	12
LDH, U/L	318	292	1834	489
C3, mg/dL	53	94	82	84
C4, mg/dL	11	13	6	12
Anti-dsDNA, IU/mL	11.0	11.1	<3	10.3

anti-dsDNA = anti-double stranded DNA, C3 = complement 3, C4 = complement 4, CRP = C-reactive protein, ESR = erythrocyte sedimentation rate, Hb = hemoglobin, Hct = hematocrit, WBC = white blood cell.

## 3 Discussion and conclusions

This patient was admitted for SLE-related immune thrombocytopenia twice before her first TTP episode. At the third event, her platelet counts were similar, but her serum total bilirubin was elevated, and neurologic symptoms developed, which was different with the previous episodes. With no abnormal findings on brain imaging, confirmation of microangiopathic hemolytic anemia on a peripheral blood smear, and plasma exchange immediately conducted, this patient achieved rapid improvement in her mental status. In addition, rituximab therapy followed, resulting in overall improvement without any sequelae or recurrence.

A review of 105 cases of SLE and TTP showed that the mortality was 12.4%, with infection and renal damage contributing to the poor outcomes in TTP-associated SLE.^[13]^ Among patients with SLE, patients with TTP (n = 24) had lymphopenia, higher SLE disease activity index score, less than 7 g/dL of hemoglobin, low levels of indirect bilirubin, and less severe thrombocytopenia than those without TTP (n = 48).^[14]^ While ADAMTS13 deficiency was associated with more severe thrombocytopenia and CNS involvement, it could indicate rapid resolution and good treatment response in SLE associated TTP.^[15]^

In a comparison of outcomes of immune-mediated TTP between patients with SLE (n = 8) and those without TTP (n = 10), mortality rates were higher, with a longer duration of treatment before remission, in patients with SLE compared in patients without SLE.^[16]^ However, another study showed the patients with primary TTP (n = 18) had more severe renal involvement compared to patients with SLE-related TTP (n = 10); clinical remission was more frequent and mortality rates were lower in patients with SLE (n = 18).^[17]^ Although it is difficult to draw firm conclusions due to the small number of study populations, it does not seem to be the case that SLE-related TTP has a worse prognosis than primary TTP.

Rituximab is a chimeric monoclonal antibody against the CD20 antigen present on B cells, leading to B cell depletion.^[^18^,^^19^^]^ Inhibition of B cell proliferation and suppression of autoantibody production by anti-CD20 antibodies was revealed to be effective in controlling acute flare-ups of SLE, including lupus nephritis and severe thrombocytopenia.^[^20^–^22^]^ Moreover, rituximab therapy that blocks anti-ADAMTS13 antibody formation has been found to be effective in acquired TTP.^[23]^ An open-label study showed that rituximab could control the disease, diminish plasma requirements, and decrease the 1-year relapse rate by diminishing the production of anti-ADAMTS13 antibodies.^[24]^ In addition, early administration of rituximab was associated with faster attainment and fewer plasma exchange, and prophylactic administration was associated with normalization of ADAMTS13 levels.^[25]^

One case report showed that a 34–year-old female presented with syncope and skin rash with hemolytic anemia and thrombocytopenia was diagnosed TTP and SLE simultaneously.^[26]^ While platelet counts were elevated after 6 days of plasma exchange and glucocorticoids, her platelet counts went down again, suggesting refractory TTP. Rituximab therapy was started, but seizures developed, so cyclophosphamide was started, and the patient saw improvement within a week. She continued rituximab administration with cyclophosphamide (500 mg). Such refractory disease requires a variety of treatment consisting of continuous plasmapheresis, rituximab and cyclophosphamide. In another case of an 8-year-old girl with acquired and autoimmune TTP, rituximab therapy has been maintained for 3 years due to recurrent episodes (7 episodes for 3 years), and low levels of ADAMTS13 activity.^[27]^ Two patients with SLE and TTP presenting neurologic symptom and renal involvement were treated with low-dose rituximab (100 mg weekly for 4 weeks) and plasmapheresis,^[28]^ and both patients recovered with no relapse for 2 years. Our patient however, experienced a recurrence after 18 months, despite 4 cycles of rituximab therapy, suggesting that the prophylactic effect of rituximab might have limitations.

In summary, we describe a patient with SLE and immune thrombocytopenia in whom TTP developed, and was treated with plasmapheresis and rituximab therapy successfully, though the patient experienced a disease relapsed after 18 months, which was controlled by the same management.

## Author contributions

**Conceptualization:** Ju-Yang Jung, Hyoun-Ah Kim.

**Data curation:** Ju-Yang Jung, Ji-Won Kim, Hyoun-Ah Kim.

**Formal analysis:** Hyoun-Ah Kim.

**Investigation:** Ju-Yang Jung.

**Methodology:** Ju-Yang Jung.

**Supervision:** Chang-Hee Suh, Hyoun-Ah Kim.

**Visualization:** Ju-Yang Jung, Ji-Won Kim, Hyoun-Ah Kim.

**Writing – original draft:** Ju-Yang Jung, Hyoun-Ah Kim.

**Writing – review & editing:** Ji-Won Kim, Chang-Hee Suh, Hyoun-Ah Kim.
